# Germanium Nanoparticles Prepared by Laser Ablation in Low Pressure Helium and Nitrogen Atmosphere for Biophotonic Applications

**DOI:** 10.3390/ma15155308

**Published:** 2022-08-02

**Authors:** Anastasiya A. Fronya, Sergey V. Antonenko, Nikita V. Karpov, Nikolay S. Pokryshkin, Anna S. Eremina, Valery G. Yakunin, Alexander Yu. Kharin, Alexander V. Syuy, Valentin S. Volkov, Yaroslava Dombrovska, Alexander A. Garmash, Nikolay I. Kargin, Sergey M. Klimentov, Victor Yu. Timoshenko, Andrei V. Kabashin

**Affiliations:** 1 MEPHI, Institute of Engineering Physics for Biomedicine (PhysBio), Kashirskoe sh. 31, 115409 Moscow, Russia; aafronya@mephi.ru (A.A.F.); svantonenko@mephi.ru (S.V.A.); nvkarpov@mephi.ru (N.V.K.); black.beast_1995@list.ru (N.S.P.); annaeremina96@mail.ru (A.S.E.); aykharin@mephi.ru (A.Y.K.); yaroslava.dombrovska@gmail.com (Y.D.); aagarmash@mephi.ru (A.A.G.); smklimentov@mephi.ru (S.M.K.); 2 Lebedev Physical Institute of the Russian Academy of Sciences, Leninskiy Pr. 53, 119991 Moscow, Russia; 3 MEPHI, Institute of Nanoengineering in Electronics, Spintronics and Photonics, Kashirskoe sh. 31, 115409 Moscow, Russia; nikargin@mephi.ru; 4 Faculty of Physics, Lomonosov Moscow State University, Leninskie Gory 1, 119991 Moscow, Russia; yvg51@bk.ru; 5 Moscow Institute of Physics and Technology (MIPT), Center for Photonics and 2D Materials, 141700 Dolgoprudny, Russia; alsyuy271@gmail.com (A.V.S.); vsv.mipt@gmail.com (V.S.V.); 6 LP3 Laboratory, Aix-Marseille University, CNRS, 13288 Marseille, France

**Keywords:** nanostructured germanium (Ge), nanoparticles, Ge nanocrystals, laser ablation, pulsed laser deposition, photoluminescence, phototherapy, theranostics

## Abstract

Due to particular physico-chemical characteristics and prominent optical properties, nanostructured germanium (Ge) appears as a promising material for biomedical applications, but its use in biological systems has been limited so far due to the difficulty of preparation of Ge nanostructures in a pure, uncontaminated state. Here, we explored the fabrication of Ge nanoparticles (NPs) using methods of pulsed laser ablation in ambient gas (He or He-N_2_ mixtures) maintained at low residual pressures (1–5 Torr). We show that the ablated material can be deposited on a substrate (silicon wafer in our case) to form a nanostructured thin film, which can then be ground in ethanol by ultrasound to form a stable suspension of Ge NPs. It was found that these formed NPs have a wide size dispersion, with sizes between a few nm and hundreds of nm, while a subsequent centrifugation step renders possible the selection of one or another NP size fraction. Structural characterization of NPs showed that they are composed of aggregations of Ge crystals, covered by an oxide shell. Solutions of the prepared NPs exhibited largely dominating photoluminescence (PL) around 450 nm, attributed to defects in the germanium oxide shell, while a separated fraction of relatively small (5–10 nm) NPs exhibited a red-shifted PL band around 725 nm under 633 nm excitation, which could be attributed to quantum confinement effects. It was also found that the formed NPs exhibit high absorption in the visible and near-IR spectral ranges and can be strongly heated under photoexcitation in the region of relative tissue transparency, which opens access to phototherapy functionality. Combining imaging and therapy functionalities in the biological transparency window, laser-synthesized Ge NPs present a novel promising object for cancer theranostics.

## 1. Introduction

Nanostructures of group IV semiconductors (Si, C, Ge and their compounds) are currently among the most popular nanomaterials in biomedical applications, including biosensing, imaging and therapies [1,2,3,4,5]. In particular, diamond nanoparticles (NPs) and other carbon-based nanostructures (graphite, SiC, etc.) have found extensive applications in bioimaging and drug delivery [1,4]. Silicon nanostructures are even more popular in biomedical tasks. Due to its natural high abundance, excellent biocompatibility [2,3] and unique biodegradability option [6,7,8], combined with the capability of generating strong PL in the visible and near-infrared [9], nanosilicon is a widely exploited material in biosensing [10], bioimaging [11,12] and various therapies based on intrinsic Si properties [13,14]. In contrast, studies of nanogermanium (nano-Ge) and its biomedical applications have been very limited so far, although being an indirect gap semiconductor (bandgap at 0.67 eV), nano-Ge is very similar to nanosilicon in terms of physico-chemical properties and is known to be a biologically friendly (when free of toxic impurities) [1,15] and water-dissolvable [16]. Furthermore, a strong light absorption by Ge nanostructures in the visible–near IR range makes them excellent candidates as sensitizers of photo-induced hyperthermia [15,17]. One of the reasons for the modest attention to nano-Ge is related to the lack of methods making possible its fabrication in a pure, uncontaminated state, suitable for employment in biosystems. Indeed, the synthesis of Ge nanocrystals is typically based on wet chemical routes [15,18,19,20,21,22,23,24], which often cause the contamination of formed NPs by toxic by-products. The second reason is related to the complexity of emission processes in Ge nanocrystals and the difficulty of implementation of PL in the window of relative tissue transparency (650–900 nm). Indeed, in most cases, the recorded PL signals from nano-Ge are in the spectral region around 300–450 nm [19,20,21,22,23], while the fabrication of Ge nanocrystals emitting the IR typically requires the involvement of complicated synthesis protocols using non-biocompatible products [24].

Laser-ablative synthesis presents an alternative “physical” approach to chemical pathways, which can solve the problem of nanomaterial contamination and render possible the control of optical response [25,26]. This method is based on a natural formation of nanoclusters under laser–material interaction, which can then be realized into either an ambient gas and deposited onto a substrate to form a thin nanostructured film [27,28,29,30,31] or onto a liquid medium to form a colloidal solution of NPs [32,33,34]. The advantage of this approach consists of ultraclean conditions of synthesis [25], which enables one to avoid all undesirable toxic impurities, typical for chemical routes. Several studies demonstrated the synthesis of Ge-based (Ge, GeO_2_, GeSi) nanocrystalline films [35,36,37,38,39,40,41] on the substrate or colloidal solutions of NPs [42,43,44] by using methods of pulsed laser ablation in gaseous or organic liquid media, respectively. Although such Ge crystallites may be of interest for optoelectronic, photovoltaic and other applications, their PL properties do not look promising for bioimaging use. Indeed, Ge nanocrystals prepared by laser ablation in gases could exhibit PL emission only around 400–500 nm [35,37], which is hardly applicable in bioimaging as it is outside the relative tissue transparency window. On the other hand, Ge nanocrystals prepared by laser ablation in organic solvents can exhibit red-shifted PL in the green [42] or red [44] range; they are typically contaminated by non-biocompatible by-products of synthesis, which complicates their biological applications.

We recently elaborated a technique of pulsed laser ablation in gases (typically, helium, He) maintained at residual pressure (pulsed laser deposition geometry) [28,29,45,46] and illustrated its efficiency in the fabrication of Si-based nanocrystalline films. In particular, when ablated in helium gas under a certain gas pressure, Si nanocrystals could exhibit PL emission around 750–800 nm with a long transient (lifetime about several μs), explained by quantum confinement of exciton states in high-quality Si nanocrystals [29,45]. We showed that after milling and water dispersion of the nanocrystals, they can be used as efficient biodegradable markers in bioimaging [45]. We later showed that PL emission can be enhanced and spectrally tuned by adding nitrogen (N_2_) gas to helium-based buffer [46]. We believe that such a strategy of using mixtures of gases can be used to fabricate Ge crystals with particular sizes and physical properties, making possible the generation of promising PL properties.

Here, we explore possibilities for the fabrication of liquid-dispersible Ge NPs exhibiting PL in the biological transparency window by using methods of pulsed laser deposition in ambient gas. We show that the required result can be achieved via the employment of pulsed laser ablation from a solid target in He-N_2_ mixtures, followed by the milling of substrate-supported nanostructured films and their dispersion in ethanol. We also show that in addition to PL emission with the peak at around 725 nm, the formed NPs exhibit a strong photoheating effect, which makes them promising candidates for biophotonic applications.

## 2. Materials and Methods

Single-crystal (100)-oriented Ge wafers (optically polished, with a diameter of 100 mm) were used as targets in laser-ablative experiments. Optically polished substrates of fused silica and crystalline Si (c-Si) were used as substrates for the deposition of Ge nanocrystals.

**Figure 1:**
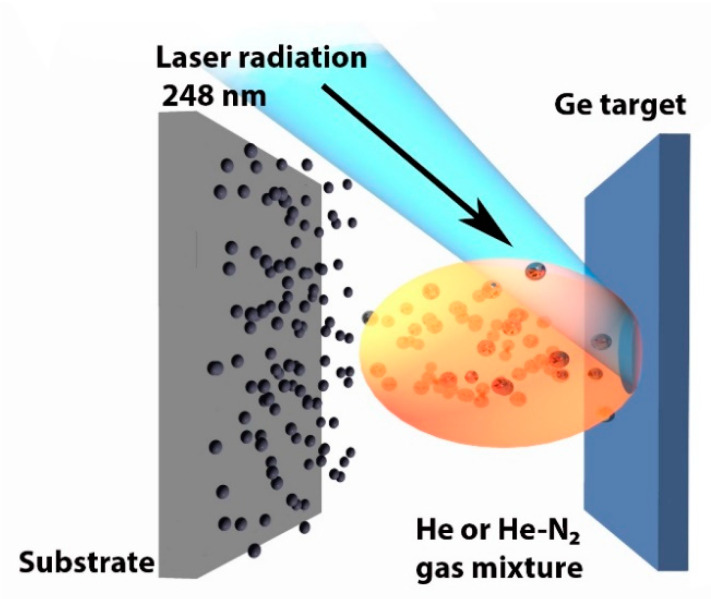
Experimental setup. A rotated Ge target is irradiated by a focused beam from UV excimer laser at the angle of 45° to initiate ablation of Ge nanoclusters in residual gases (He or He-N_2_ mixtures). The nanoclusters are deposited on a substrate located 2 cm from the target surface.

The target was ablated similarly to our previous study [46] using a fully automated pulsed laser deposition system (MBE-2000, PVD Products, Wilmington, MA, USA). This system employs a KrF excimer laser COMPexPro 110 (Coherent/Lambda Physik) operating at the wavelength of 248 nm, laser pulse length of 30 ns and repetition rates of 1–105 Hz. The output laser energy was 120 mJ, corresponding to pulse energy density of 3800 mJ/cm^2^. The repetition rate was 10 Hz. The selection of UV laser radiation ensures its strong absorption by c-Ge and the transparency of formed plasma plume to oncoming radiation. Laser power was kept constant during the deposition process (variations less than 10%). All experiments were carried out in a vacuum camera, which was initially pumped out down to a pressure of 10^−7^ Torr and then filled with buffer gases (helium (He) or helium + nitrogen (N_2_)), maintained at residual pressures from 1 to 5 Torr. The target was constantly rotated and irradiated at the incident angle of 45° to initiate ablation of material perpendicularly to the target surface, as shown in Figure 1. The laser beam spot surface was 3 mm^2^. The material was then deposited on a substrate placed 2 cm from the target, which was selected as the optimal distance for a selected laser pulse energy and the range of pressures. The ablation process led to the formation of Ge-based films on the substrate. Parameters of four representative samples deposited at different pressures and proportions of He and N_2_ are given in Table 1.

A Scanning Electron Microscopy (SEM) system (TESCAN MAIA 3) operating at 0.1–30 kV was used to study the morphology of laser-ablated films. Structural analysis of the formed NPs was carried out by using a Jeol JEM-2100 transmission electron microscope (TEM) with a resolution of 0.19 nm. Crystallinity of the films was studied by means of X-ray diffraction (XRD) and Raman spectroscopy using a Radian XDR spectrometer (Expert Center Ltd., Moscow, Russia) and a confocal micro-Raman spectrometer (Confotec MR350, SOL Instr.) under laser excitation at 632.8 nm, respectively. The same spectrometer and excitation source were also used to measure PL in the range of 650–1100 nm. PL measurements under laser excitation at 351 and 405 nm were also carried out using a Mightex HRS CCD-spectrometer (Mightex Systems, Toronto, ON, Canada). Measurements of the optical extinction coefficient of Ge NPs dispersed in ethanol were carried out using a standard spectrophotometer in the spectral range from 250 to 1000 nm. Photo-induced heating of ethanoic suspension of Ge NPs was measured using a FLIR C3 thermal imager with an accuracy of 0.1 K. The measurements were carried out for 0.5 mL of the suspension in a polystyrene cuvette under irradiation with unfocused radiation of two semiconductor lasers with power of 0.2 W, beam size about 5 mm and wavelengths of 650 and 810 nm.

## 3. Experimental Results and Discussion

### 3.1. Structural Properties

When deposited on silicon substrates, Ge-based films looked predominantly black or dark-gray colored, which agrees with previous studies of Ge-based nanostructured films [42,43,44]. Samples deposited at relatively high He pressures (5.0 Torr) had a whitish tint, suggesting the formation of germanium oxide (GeO_2_) fraction. As follows from typical SEM images of Ge films (Figure S1 of Supplementary Materials), all deposited Ge-based films had a porous texture similar to laser-ablated Si-based films [29,45]. The porosity increased when the pressure of ambient gas was increased from 1 Torr to 5 Torr, which also agrees with a porosity evolution of previous data on laser-ablated Si-based nanostructured films. A detailed analysis of the films showed that they are composed of a dense stack of Ge nanocrystals embedded in a single matrix. EDX analysis of deposited films confirmed that they are mostly composed of germanium, which provided a largely dominating signal in EDX spectra (Figure S2). All prepared samples exhibited EDX signals associated with oxygen, suggesting the presence of germanium oxide (GeO_x_, 1 < x < 2). We suppose that the formation of germanium oxide took place during a post-fabrication oxidation of nanostructured germanium films under their aging in ambient atmospheric air. The ratio of oxygen to germanium was the highest for the Ge5.0 sample, suggesting that a high porosity maximizes the surface area for oxidation phenomena. The sample deposited in the mixture of He and N_2_ (Ge2.5/2.5) additionally exhibited a signal associated with nitrogen, evidencing the formation germanium nitride or oxynitride compounds. It should be noted that EDX signals related to Si corresponded to the c-Si substrate used in our experiments.

**Figure 2:**
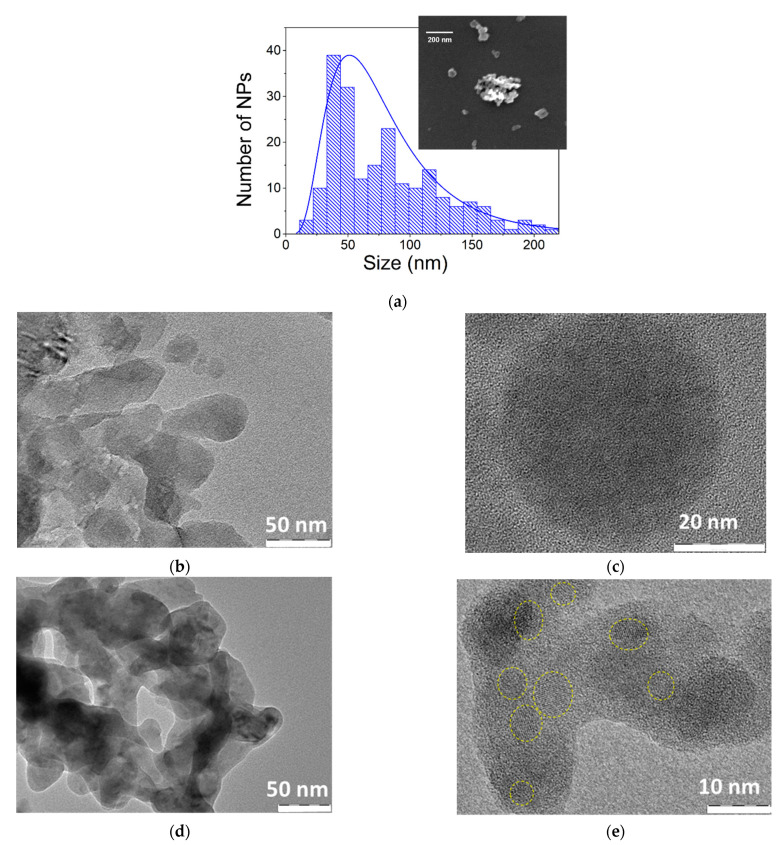
(**a**) Typical SEM image and corresponding size distribution of Ge NPs obtained by grinding of laser-ablated Ge nanostructured films (sample Ge2.5/2.5 NPs) and dispersed in ethanol after the centrifugation. The size distribution was calculated using a neural network analysis [47]. Typical TEM images of Ge NPs obtained by grinding of Ge nanostructured films and dispersed in ethanol: sample Ge 5.0 NPs (**b**,**c**) Ge2.5/2.5 NPs (**d**,**e**). Yellow dashed circles in panel (**e**) highlight locations of Ge nanocrystals.

Biomedical applications imply the employment of colloidal NPs solutions rather than nanostructured films. To fabricate Ge-based NPs, we applied a technique of ultrasound grinding similar to our previous studies related to Si NPs [45,46]. Since Ge is not stable against dissolution in water [16], we used ethanol to avoid the dissolution process during NP storage. The films were ground from the substrate and sonicated in ethanol (95%) under 20 kHz ultrasound for 30 min. A part of the formed suspension was centrifuged for 5 min at 5000 g and the supernatant was used for further analysis as a small size fraction of Ge NPs. As shown in Figure 2a, NPs from the small size fraction presented agglomerates of many crystals with sizes ranging from a few nm to 200 nm and a mean size of about 50 nm (size of NPs in original sample could reach μm range).

TEM investigation confirmed conclusions from SEM measurements on the size distribution of Ge NPs. Figure 2 shows high-resolution TEM images of typical Ge NPs obtained by grinding of Ge nanostructured films in ethanol for sample Ge5.0 (b,c) and Ge2.5/2.5 (d,e). One can see that most NPs consist of coagulated aggregations of small Ge structures of arbitrary shape with sizes between 20 and 50 nm. Furthermore, the analysis of high-resolution TEM images of NPs produced from He/N_2_ mixture evidences that their structure is composed of smaller nanocrystals with sizes of 5–7 nm, which are embedded in an amorphous germanium matrix (see Figure 2e, as highlighted by yellow marker). The presence of such small nanocrystals promises possible generation of the quantum confinement regime, which can influence PL properties of the prepared Ge NPs. It should be noted that our measurements did not resolve Ge crystalline planes from NPs in the case of sample Ge5.0 (Figure 2c), which could be explained by highly defected crystal structure or the domination of an amorphous phase.

**Figure 3:**
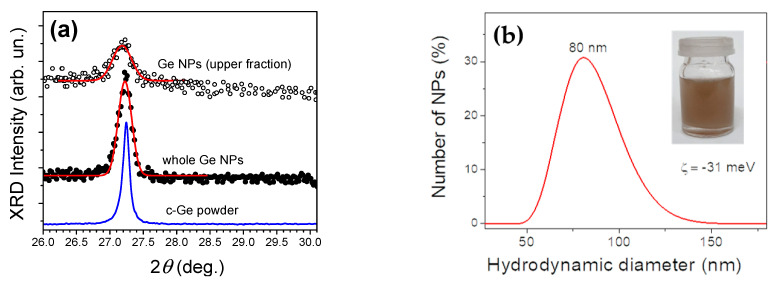
(**a**) XRD spectra in the vicinity of the (111) crystalline lattice of c-Ge from dried suspensions of Ge2.5/2.5 NPs (open and dark circles) after applying Gaussian function fits (red lines) and that of a c-Ge powder (grey line); the middle and upper spectra correspond to Ge NPs from the integral (whole) ensemble and the small size fraction (upper fraction), respectively. (**b**) Size distribution of Ge NPs from the DLS data. Inset shows the photographic image of a vessel with Ge NP suspension in ethanol.

To examine the structural properties of Ge NPs, their suspensions were dropped on a metal substrate and dried. The powders were then examined by a variety of techniques, including XRD, DLS and Raman spectroscopy. Our tests showed that XRD spectra of dried suspensions of Ge2.5/2.5 NPs corresponded to well-known data for crystalline Ge [18]. This fact is illustrated by the presence of an XRD peak at 27.3°, corresponding to (111)-crystallographic plane of Ge, as one can see from the comparison of spectra of Ge NPs and that of c-Si powder measured at the same angular resolution (blue line in Figure 3a). No characteristic peaks of crystalline GeO_2_ phases (26.5°, 28.7° and 29.8°) were found, which evidenced an amorphous nature of the oxide shell of Ge NPs. The XRD peak of Ge2.5/2.5 NPs exhibits broadening of about 0.25° and 0.35° in the case of samples, corresponding to the integral NP ensemble and the small size fraction, respectively. These broadening values were analyzed using the Scherrer equation to estimate the X-ray coherent scattering dimensions, which are known to characterize the mean size of Ge nanocrystals. Such an estimation provides the mean size of about 40 and 30 nm for the integral sample and fine fraction, respectively, which roughly corresponds to our estimations from TEM observations.

Figure 3b shows a DLS spectrum generated by the integral (whole) ensemble of Ge2.5/2.5 NPs suspension in ethanol. Here, the average hydrodynamic diameter of Ge NPs is close to 80 nm, which is 2-fold larger compared with crystallite size assessed from TEM and XRD data. Such a difference can be explained by the contribution of agglomerates or the presence of a relatively thick amorphous oxide shell on the surface of NPs. Zeta-potential of Ge NPs in ethanol suspension (sample Ge2.5/2.5) was found to be about −31 eV. While the negative sign of the latter can be explained by the predomination of hydroxyl radical (OH^−^) coating of Ge NPs, a relatively large absolute value of zeta-potential corresponds to the good stability of NPs in suspension.

**Figure 4:**
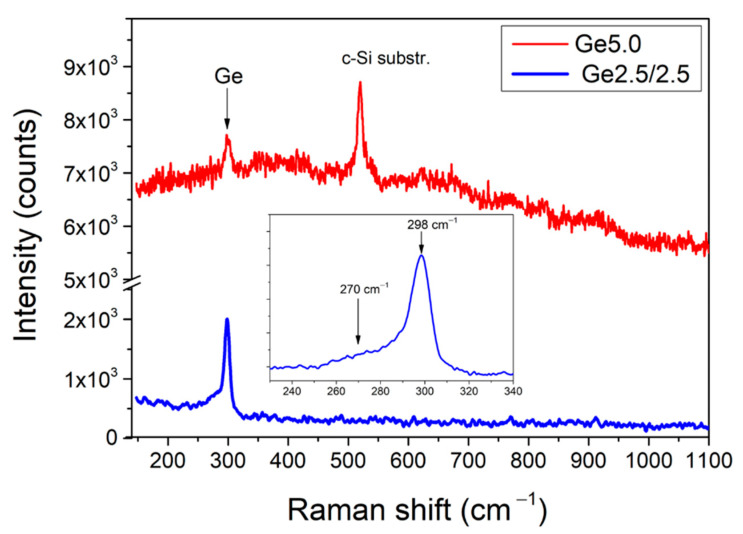
Raman spectra from Ge NPs obtained by grinding of laser-ablated Ge-based nanostructured films. Red curve (upper spectrum) and blue curve (lower spectrum) correspond to samples Ge5.0 and Ge2.5/2.5, respectively. Inset shows a selected region of the Raman spectrum of the dried small size fraction of Ge2.5/2.5 NPs.

The Raman spectroscopy of laser-ablated Ge films evidences their nanocrystalline structure, which is justified by the presence of a Raman peak at 298–299 cm^−1^ (Figure S3a). While the position of this peak is identical for all samples, its intensity is different with the weakest signal for Ge5.0, followed by increasingly higher intensity for samples Ge2.0, Ge1.0 and maximal intensity for Ge2.5/2.5, suggesting the increase in crystallinity with the decrease in He pressure and the addition of N_2_ as the buffer gas. The Raman examination of dried suspensions of Ge NPs confirms the same tendency in the evolution of crystallinity of these samples. As an example, dried suspensions of Ge5.0 and Ge2.5/2.5 NPs exhibit the same peak around 298–299 cm^−1^ associated with the presence of nanocrystalline Ge, but the intensity of this peak is significantly higher for Ge2.5/2.5 NPs (Figure 4), suggesting its higher crystallinity. Notice that this experimental result agrees with the TEM data (Figure 2b–e) that also evidenced the nanocrystallinity of Ge2.5/2.5 NPs. As shown in the inset of Figure 4, the Raman peak at 298–299 cm^−1^ for the Ge2.5/2.5 sample is accompanied by a broad shoulder at about 280 cm^−1^, which can be explained by the presence of either very small crystalline NPs or some amorphous Ge. The presence of this shoulder is also resolvable for samples prepared in pure He ambient (Figure S3a). It should be noted that the supposition on the presence of a fraction amorphous Ge fraction was also confirmed by results of TEM measurements (Figure 2).

### 3.2. Photoluminescence of Ge Samples

As shown in Figure S3b, as-prepared laser-ablated films deposited in the atmosphere of pure He exhibit PL spectra centered at 450–470 nm, which agrees with previous results on laser-ablative formation of Ge films in PLD geometry [35,37]. The intensity of this peak is maximal for the sample Ge5.0, followed by Ge2.0 and Ge1.0. The emission around 450–470 nm is usually attributed to a radiative transition at electronic defect-related states in GeO_x_ [15]. We suppose that such a band could be due to the formation of a germanium oxide-based upper layer after the exposition of films to air. This hypothesis is supported by the fact that maximal signals of PL were recorded for the Ge5.0 sample, which was characterized by the highest oxidation degree, probably due to higher porosity, as confirmed by color of the prepared films and EDX data (Figure S2), as well by the Raman spectroscopy. On the other hand, the peak around 450–470 nm was very weak in the case of the sample Ge2.5/2.5 (Figure S3b), suggesting that the presence of N_2_ could somehow passivate the surface of nanocrystals and thus minimize subsequent oxidation and the formation of GeO_x_ defects. On the other hand, we could resolve a weak PL tail in the near-IR from this sample. A similar tendency in the evolution of PL signals was observed for dried suspensions of Ge NPs from all these samples (Figure S4).

**Figure 5:**
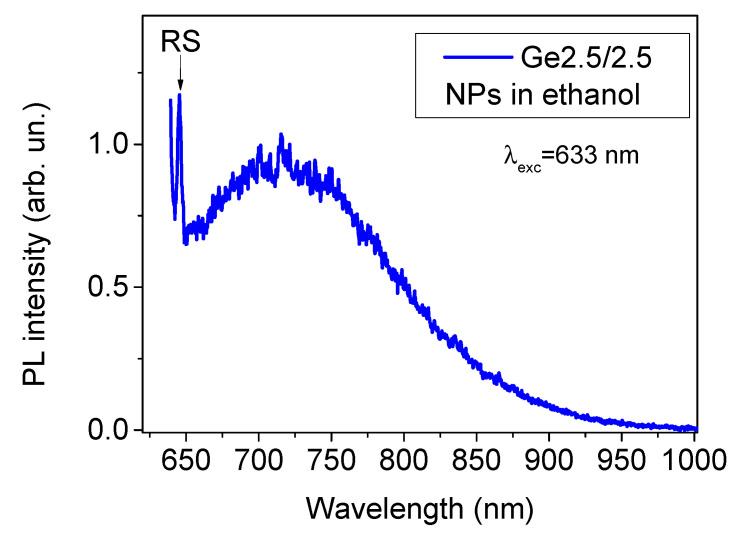
Photoluminescence spectrum from small size fraction of Ge NPs from Ge2.5/2.5 sample under 633 nm laser excitation. RS signature shows the position of Raman scattering peak at 298–299 cm^−1^ associated with Ge nanocrystals.

Since the sample Ge2.5/2.5 demonstrated the lowest level of GeO_x_ defect-related PL and demonstrated signs of the PL emission in near-IR, we selected this sample for a further detailed examination. Using a centrifugation step, we separated the small size fraction of these NPs and assessed their properties under 632.8 nm excitation matching the optical transparency window. Surprisingly, we observed quite different PL emission, namely, a broad band in the spectral region from 650 to 1000 nm with the maximum around 725 nm (Figure 5). It is important that such a PL emission was accompanied by the Raman scattering peak (RS in Figure 5), suggesting that the PL was due to the radiative recombination in Ge crystals. Based on the position of the peak and the presence of RS crystal signature, such a PL emission can be associated with a radiative recombination of excitons confined in small Ge nanocrystals with sizes below 10 nm [48] or/and of charge carriers trapped on electronic states in the oxide shell of NPs. Since PL in red-IR spectral region was observed only for the Ge2.5/2.5 samples, one can suppose a critically important role of nitrogen in passivation of the surface of Ge nanocrystals, conditioning the appearance of this emission. Of note, the effective Bohr radius of Ge is about 25 nm [18] that ensures the quantum confinement effect in the prepared Ge NPs.

### 3.3. Light Absorbance and Photoheating by Ge-NPs

**Figure 6:**
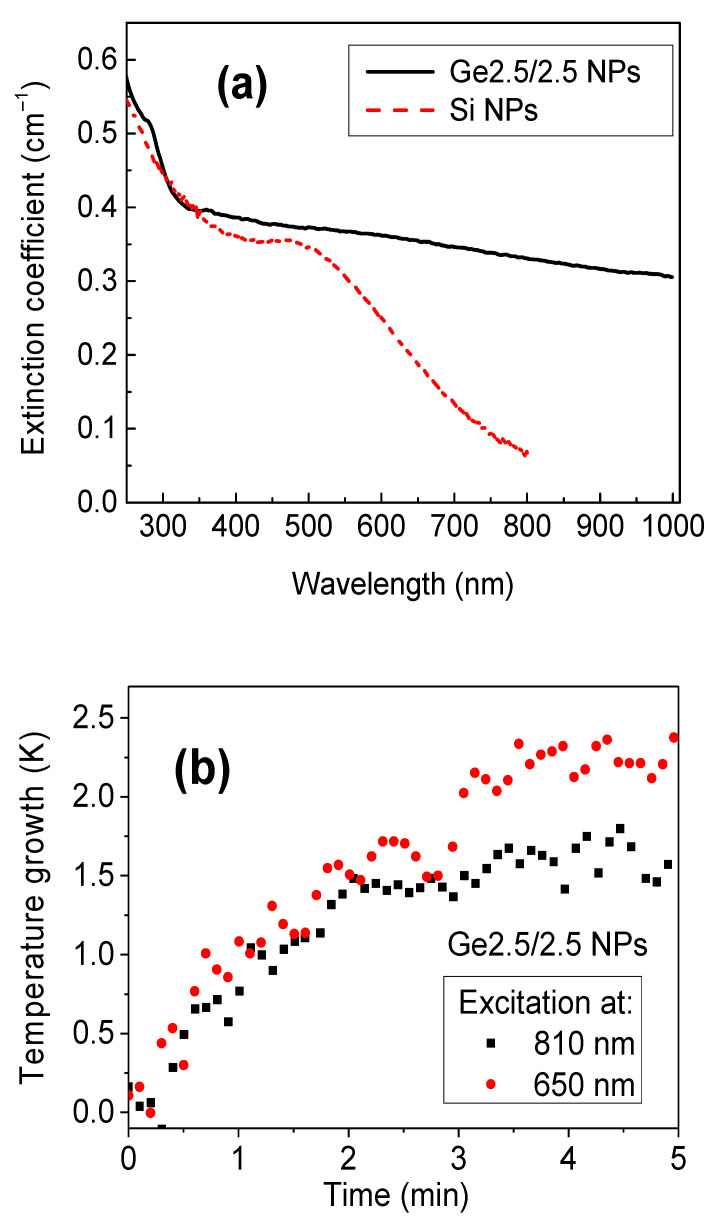
(**a**) Extinction spectra from a suspension of laser-synthesized Ge NPs (Ge2.5/2.5 sample) at concentration of 0.1 mg/mL (black curve) and that of laser-synthesized Si NPs at the same concentration (red dashed curve) from Ref. [46]; (**b**) temperature growth of the suspension of Ge NPs (sample Ge2.5/2.5) under photoexcitation with the wavelengths of 650 (red circles) and 810 (black squares) nm.

Figure 6a shows an extinction spectrum for ethanoic suspension of Ge NPs from the Ge2.5/2.5 NPs sample. One can see that the extinction intensity experience a sharp drop in the UV range and then the evolution of extinction coefficient smoothens to slowly decrease up to wavelengths of 1000 nm and higher. Such a behavior drastically contrasts with early reported extinction spectra of suspensions of laser-ablated Si NPs having similar concentrations [49]. Indeed, as shown in Figure 6a, the extinction spectrum curve of Ge NPs is much higher compared to the relevant parameter in the case of Si NPs in a wide spectral region ranging from UV to near-IR, while the difference is especially significant in the region 630–950 nm, which matches the biological transparency window. Such a high extinction coefficient of Ge NPs is obviously due to particular optical properties of Ge nanocrystals [48] forming the core of laser-ablated NPs. Finally, the extinction is obviously affected by the light scattering, which is dependent on the size distribution of Ge NPs.

Since Ge NPs have a very high extinction coefficient in the region of relative tissue transparency (630–950 nm), it is logical to suppose that they can have high absorption, which can open up access to their use as sensitizers of local photon-induced hyperthermia of malignant tissues, termed as phototherapy. To verify this supposition, we carried out a series of tests to assess the photo-heating efficiency of suspensions of laser-ablated Ge NPs from the Ge2.5/2.5 sample. As shown in Figure 6, we recorded a similar temperature growth under irradiation with semiconductor lasers emitting at 650 and 810 nm. The observed heating rate of the order of 1 K/min for the irradiation at relatively low power and wavelength in the near-IR window of the maximal transparency of biological tissue appears promising for photo-hyperthermia applications, as it was reported for Si NPs [49].

## 4. Discussion

Thus, we showed that methods of pulsed laser deposition in ambient He gas or He-N_2_ gas mixtures maintained at residual pressures of 1–5 Torr can be used to form Ge-based nanostructured films composed of Ge nanocrystals, which can then be ground by ultrasound to form colloidal nanoparticle solutions (ethanol solutions in our case). It is known that the action of laser radiation on a solid target leads to ablation of material in the form of nanoscale clusters with sizes ranging from a fraction of nm to a few nm, while their subsequent growth is strongly affected by collisions with atoms/molecules of a residual buffer gas [50,51]. First, the nanoclusters enter the zone of gas ions, produced by the ionization of gas atoms/molecules by radiation from hot laser plasma. The collisions of nanoclusters with hot gas ions can lead to their heating up to 1000 K and higher, which creates conditions for subsequent formation of high-quality crystals [45]. After the exit of nanoclusters from the plasma zone, they continue to collide with cold gas atom/molecules, which leads to their fast cooling and growth due to coalescence effects, followed by their crystallization. Being partially/fully crystallized, the nanoclusters come to the substrate surface as coagulated aggregations, forming porous layers. In this case, higher pressure of the ambient gas leads to a faster cooling of nanoclusters and a higher porosity of formed layers [29]. As follows from our experiments, the formed Ge-based nanostructured layers are mostly crystalline under the selected range of ambient pressures, but some amorphous fraction can be present. While working in a pure He atmosphere, higher crystallinity was observed for samples deposited at a relatively low pressures (1 and 2 Torr), while the sample deposited in the He+N_2_ mixture (Ge2.5/2.5) exhibited the higher crystallinity. Such an evolution of crystallinity can be explained by the interplay of heating and cooling steps in the case of pure He gas ambient, and by a larger molecular weight of N_2_ and its lower ionization potential in the case of He+N_2_ mixtures. Further evolution of the structure and electron properties of the films obviously took place after a subsequent exposition of the films to ambient air. During this stage, a thin layer of germanium oxide (GeO_x_) was formed on the surface of Ge nanocrystals. As follows from the color of formed films and EDX data, the thickness of the oxide layer increased with the increase in He pressure during the film deposition, which was obviously due to higher porosity of films formed under high He pressures. Our data also showed that the passivation of Ge nanocrystals during the deposition of films in He-N_2_ mixtures somehow decreased the impact of oxidation phenomena.

We believe that both laser ablation and subsequent oxidation steps play an important role in the formation of luminescent centers. The PL band around 450–470 nm is typically related to defect centers in the GeO_x_ matrix [15,16,17,18,19,20,21,22,23,24]. This supposition is confirmed by the correlation of recorded intensity of this peak with the oxidation degree of samples prepared under different pressure of He gas. Indeed, the intensity of this peak was maximal for samples Ge5.0 (Figure S3b), which were characterized by the highest degree of oxidation (Figure S2). As follows from Figure S3, the addition of N_2_ could somehow minimize the impact of such a defect-related PL, which was probably related to a particular passivation of nanocrystal surface by nitrogen. On the other hand, such a passivation obviously helped to form luminescent centers providing the emission in near-IR (Figure 5), which could be attributed to the radiative recombination of photoexcited charge carriers in the smallest Ge nanocrystals or/and on electronic states at the interface between the Ge nanocrystal core and its oxidized shell [48]. To the best of our knowledge, this is the first observation of PL in the region of relative tissue transparency from Ge-based films prepared by laser ablation. Another important result consists of the observation of strong absorption in the near-IR range and the demonstration of a photoheating effect for suspensions of Ge-based NPs, which opens access to the use of these NPs as sensitizers of phototherapy based on local overheating of cancer cells, similar to Si NPs [49]. It is important that the light absorption by laser-synthesized Ge NPs is superior to that of Si NPs (Figure 6a), which promises a better therapeutic outcome under the same applied laser power.

The fabrication of Ge nanoparticles combining PL in the window of relative tissue transparency and the photothermal effect opens up avenues for the development of novel theranostic (therapy + diagnostics) agents on the basis of laser-synthesized Ge nanoparticles that can be targeted to tumors via enhanced permeability and retention (EPR) effect or the use of tumor-specific targeting molecules. In this case, the photoluminescence signal can be used to visualize the localization of the tumor area, while the photoheating effect will serve to eliminate cancer cells via hyperthermia. As another opportunity for biomedical applications, we see the use of Ge-based layers as matrices for bioimmobilizations in plasmonic biosensing [52,53], similar to Si-based layers [54]. To excite surface plasmons over such a matrix, a silicon-based prism having high refractive index (>3) is typically required [55]. Our further studies will address the assessment of Ge-based NPs in biological models to clarify cytotoxicity, imaging and phototherapy abilities.

## 5. Conclusions

We demonstrated a “green” (compared to chemical methods) synthesis of suspensions of Ge nanoparticles exhibiting PL in the infrared window of relative biological transparency by applying methods of laser ablation in pulsed laser deposition geometry, followed by ultrasound grinding of formed nanocrystalline films. The fabricated NPs have a wide size dispersion, but a subsequent centrifugation step renders possible size selection of NPs. Structural characterization of NPs by a variety of methods, including Raman spectroscopy, XRD, TEM, SEM and DLS, showed that they are composed of aggregations of Ge nanocrystals, covered by oxide shells. We also found that photoluminescent properties of formed Ge nanoparticles are strongly affected by the gas composition during the laser ablation process. While Ge NPs prepared in the atmosphere of pure He at low pressure exhibited photoluminescence around 450 nm attributed to defects in the germanium oxide shell, the addition of N_2_ to ambient He led to the appearance of a PL band around 725 nm under 633 nm excitation, which could be due to the quantum confinement in small Ge nanocrystals. We also showed that the suspensions of Ge NPs can be strongly heated under photoexcitation in the region of relative tissue transparency, which promises an attractive phototherapy functionality. The demonstrated ability of laser-synthesized Ge NPs to combine the room temperature PL and photothermal response in the window of relative biological transparency makes them a promising object for theranostic applications. Such an expectation is alimented by a water-dissolvability of Ge nanostructures, promising a fast biodegradability option.

## Figures and Tables

**Table 1:** Samples of Ge nanocrystalline films prepared by PLD.

Sample	P_He_, Torr	P_N2_, Torr	Total Pressure, Torr
Ge1.0	1.00	0.00	1.00
Ge2.0	2.00	0.00	2.00
Ge5.0	5.00	0.00	5.00
Ge2.5/2.5	2.50	2.50	5.00

## Data Availability

Not applicable.

## Supplementary Materials

The following are available online at https://www.mdpi.com/article/10.3390/ma15155308/s1, Figure S1: SEM images of selected regions of the PLD-deposited Ge films prepared at different gas mixtures, Figure S2: EDX elemental analysis of Ge5.0 (a) and Ge2.5/2.5 (b) films, Figure S3: (a) Raman spectra of PLD-deposited Ge films on c-Si substrate under excitation at 632.8 nm; (b) PL spectra of the same films under laser excitation at 351 nm. Figure S4: PL spectra of dried suspensions of Ge NPs under laser excitation at 351 nm.
