# Supplementary material for: Germanium Nanoparticles Prepared by Laser Ablation in Low Pressure Helium and Nitrogen Atmosphere for Biophotonic Applications
Source: Materials (Basel). 2022 Aug 2;15(15):5308. doi: 10.3390/ma15155308 (PMC9369467; doi:10.3390/ma15155308)
Supplement: Supplementary file 1 [file materials-15-05308-s001.zip › materials-1774691-supplementary.pdf]

Supplementary Materials

Germanium Nanoparticles Prepared by Laser Ablation in Low Pressure Helium and Nitrogen Atmosphere for Biomedical Applications

Anastasiya A. Fronya[1,2], Sergey V. Antonenko[1,3], Nikita V. Karpov[1], Nikolay S. Pokryshkin[1,4], Anna S. Eremina[1], Valery G. Yakunin[4], Alexander Yu. Kharin[1], Alexander V. Syuy[5], Valentin S. Volkov[5], Yaroslava Dombrovska[1], Alexander A. Garmash[1], Nikolay I. Kargin[3], Sergey M. Klimentov[1], Victor Yu. Timoshenko[1,2,4], Andrei V. Kabashin[6]

[1] MEPHI, Institute of Engineering Physics for Biomedicine (PhysBio), Kashirskoe sh. 31, 115409 Russia; AAFronya@mephi.ru (A.A.F.); black.beast_1995@list.ru (N.S.P.); annaeremina96@mail.ru (A.S.E.); AYKharin@mephi.ru (A.Y.K.); NVKarpov@mephi.ru (N.V.K.); yaroslava.dombrovska@gmail.com (Y.D.), SSGarmash@mephi.ru (A.A.G.), SMKlimentov@mephi.ru (S.M.K.), vtimoshe@gmail.com (V.Y.T.)

[2] Lebedev Physical Institute of the Russian Acad. Sci., Leninskiy Pr. 53, 119991 Moscow, Russia; nastya708@yandex.ru (F.A.A.), timoshenkovy@lebedev.ru (V.Y.T.)

[3] MEPHI, Institute of Nanoengineering in Electronics, Spintronics and Photonics, Kashirskoe sh. 31, 115409 Moscow, Russia; SVAntonenko@mephi.ru (S.V.A.), NIKargin@mephi.ru (N.I.K)

[4] Lomonosov Moscow State University, Faculty of Physics, Leninskie Gory 1, 119991 Moscow, Russia; black.beast_1995@list.ru (N.S.P.); yvg51@bk.ru (Y.V.G.), timoshen@physics.msu.ru (V.Y.T.)

[5] Moscow Institute of Physics and Technology (MIPT), Center for Photonics and 2D Materials, 141700 Dolgoprudny, Russia; alsyuy271@gmail.com (A.V.S.), vsv.mipt@gmail.com (V.S.V.)

[6] Aix Marseille Univ, CNRS, LP3, Campus de Luminy, Case 917, 13288 Marseille, France; andrei.kabashin@univ-amu.fr (A.V.K.)

1. SEM and EDX

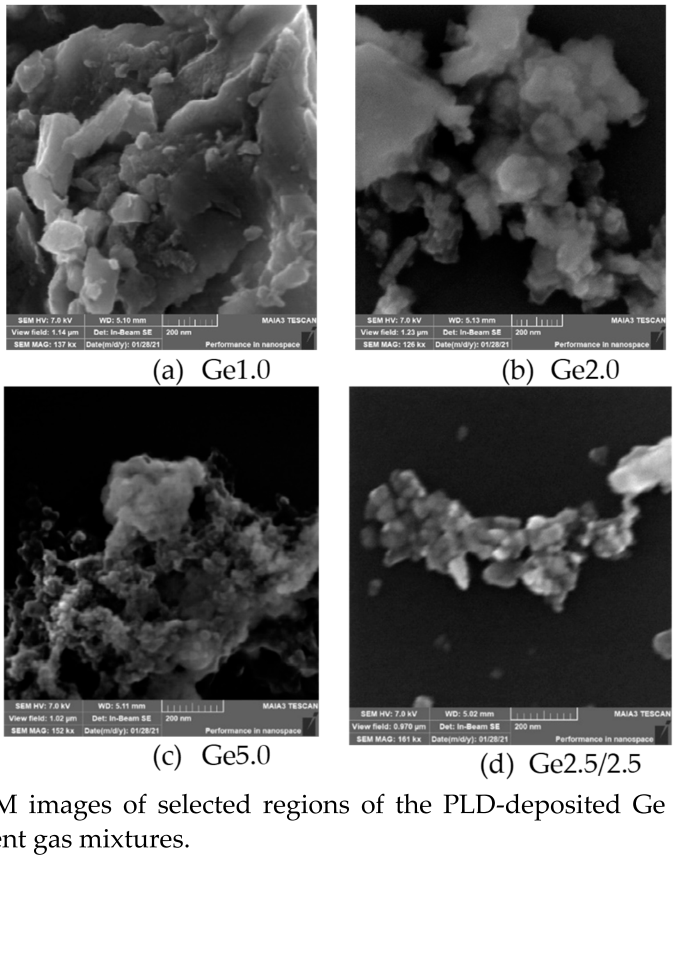

Figure S1. SEM images of selected regions of the PLD-deposited Ge films prepared at different gas mixtures.

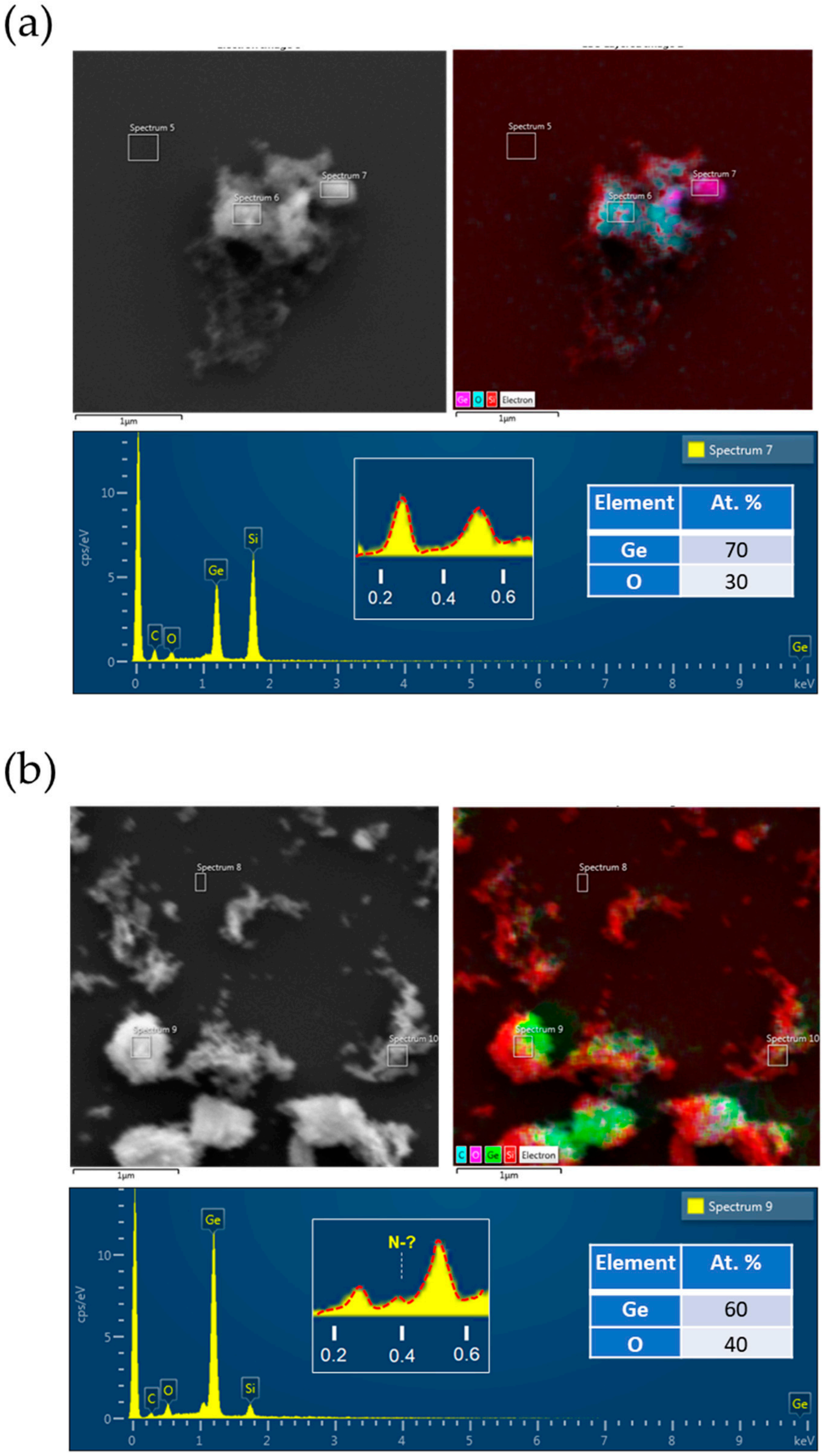

Figure S2. EDX elemental analysis of (a) Ge5.0 and (b) Ge2.5/2.5 films.

2. Raman and PL spectra

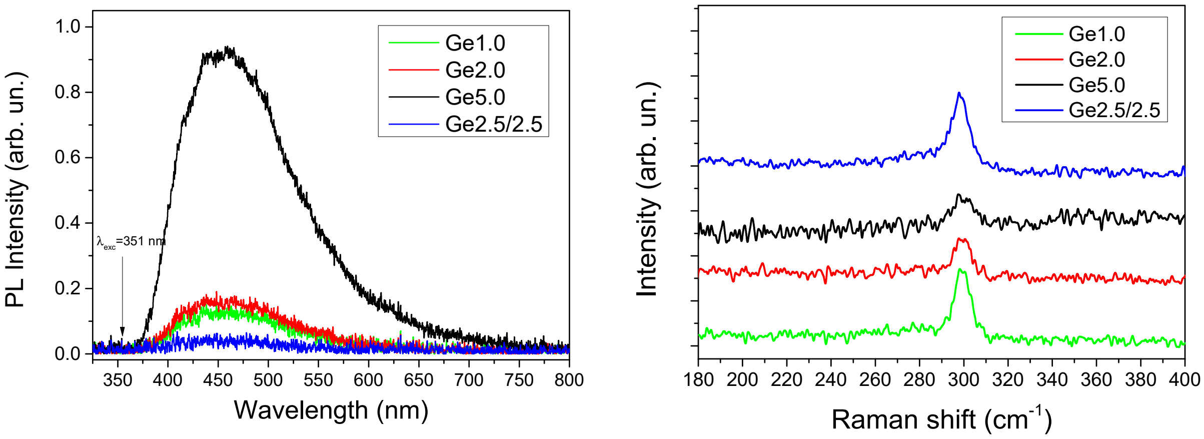

(a) **(b)**

Figure S3. (a) Raman spectra of PLD-deposited Ge films on c-Si substrate under excitation at 632.8 nm; (b) PL spectra of the same films under laser excitation at 351 nm.

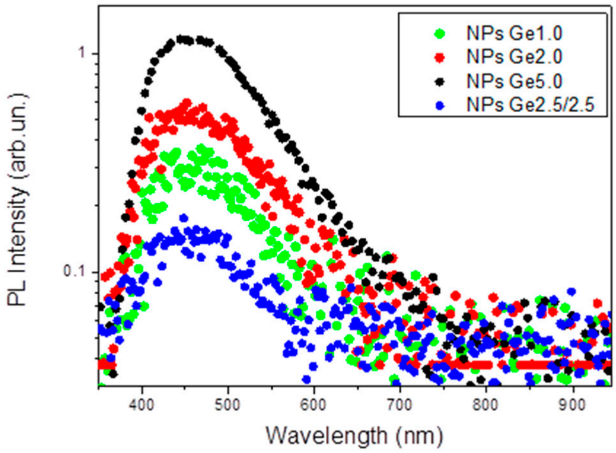

Figure S4. PL spectra of dried suspensions of Ge NPs under laser excitation at 351 nm.
